# Cytotoxicity and Pro-Apoptotic Activity of 2,2´-Bis[4,5-bis(4-hydroxybenzyl)-2-(4-hydroxyphenyl)cyclopent-4-en-1,3-dione], a Phenolic Cyclopentenedione Isolated from the Cyanobacterium Strain *Nostoc* sp. str. Lukešová 27/97

**DOI:** 10.3390/molecules16054254

**Published:** 2011-05-23

**Authors:** Jan Vacek, Jan Hrbáč, Jiří Kopecký, Jitka Vostálová

**Affiliations:** 1 Department of Medical Chemistry and Biochemistry, Faculty of Medicine and Dentistry, Palacký University, Hněvotínská 3, 775 15 Olomouc, Czech Republic; 2 Department Physical Chemistry, Faculty of Science, Palacký University, tř. 17.listopadu 12, 771 46 Olomouc, Czech Republic; 3 Department of Autotrophic Microorganisms, Institute of Microbiology, Academy of Sciences of the Czech Republic, Opatovický mlýn, 379 81 Třeboň, Czech Republic

**Keywords:** polyphenol, cyclopentenediones, cytotoxicity, apoptosis, necrosis

## Abstract

The cytotoxicity of the polyphenol 2,2´-bis[4,5-bis(4-hydroxybenzyl)-2-(4-hydroxyphenyl)cyclopent-4-en-1,3-dione], nostotrebin 6 (NOS-6), was tested under *in vitro* conditions using mouse fibroblasts (BALB/c cells). Identification of NOS-6 and its uptake into fibroblasts was examined by multi-stage mass spectrometry analysis with the following fragmentation pattern: MS (*m/z*) [M+H]^+^ 799.1 → MS^2^ 399.1 → MS^3^ 305.1 → MS^4^ 277.1. Using several cell viability assays, the IC_50_ of NOS-6 after 24 h incubation was found to be 8.48 ± 0.16/12.15 ± 1.96 µM (neutral red/MTT assay) which was higher than that of doxorubicin. It was found that NOS-6 is capable of inducing both types of cell death, apoptosis and necrosis in a dose-dependent manner. The biological activities of the cyclopentenediones and preliminary data on NOS-6 cytotoxicity are discussed.

## 1. Introduction

Cyanobacteria and algae are producers of secondary metabolites and a number of these are phenolic compounds that induce diverse biological effects in mammals [1]. Of these phenolics, phenolic acids and their esters [2,3], a large group of phenols derived from phloroglucinol (phlorotannins) [4] and halogenated and sulphated derivatives of phenols [5] have been found (reviewed in [1,6]). Soluble algal phenolics may have adaptive functions in interaction with other organisms or with the abiotic environment [7]. Secondary metabolites, including phenolic compounds, in cyanobacteria and algae and their biological activities were reviewed in [8].

**Figure 1:**
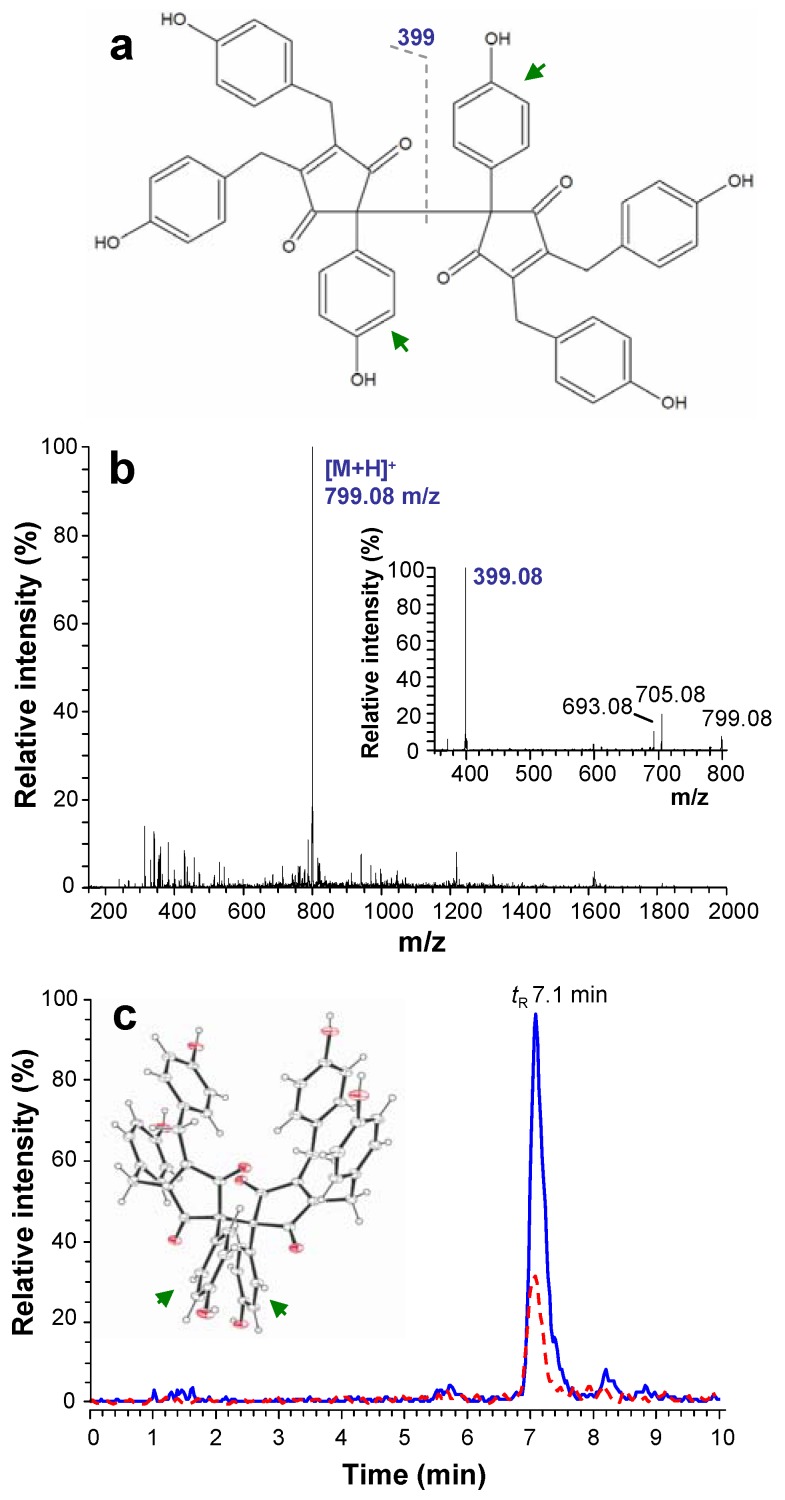
**(a)** Chemical structure and **(b)** full MS spectrum of NOS-6. **(c)** Chromatographic records of NOS-6 in homogenate prepared from mouse fibroblasts (BALB/c cells), after 4 h cultivation with 1 μM NOS-6 (dashed line: NOS-6 model standard solution; 1 μg∙mL^−1^). The MS^2^ spectrum and 3-D molecular structure of NOS-6 are shown in insets of panel **b** and **c**, respectively.

One interesting polyphenolic compound that has been recently identified in cyanobacteria is the fully substituted 2,2´-bis(cyclopent-4-en-1,3-dione) nostotrebin 6 (NOS-6) [9,10,11] (Figure 1a). NOS-6 has been isolated from cyanobacterium *Nostoc* sp. strain Lukešová 27/97 [11] and subsequently characterized using nuclear magnetic resonance (NMR), X-ray crystallography and mass spectrometry (MS) [10]. X-ray crystallographic analysis of NOS-6 has shown its structure to be 2,2´-bis[4,5-bis(4-hydroxybenzyl)-2-(4-hydroxyphenyl)cyclopent-4-en-1,3-dione].

NOS-6 is an effective acetylcholinesterase and butyrylcholinesterase inhibitor under *in vitro* conditions [9,11]. Other biological activities, evaluation of cytotoxicity and cellular interactions of NOS-6 have not been described until now. On the other hand, the various biological activities have been shown for cyclopentenediones (two cyclopentenediones are integral part of the NOS-6 molecule) and their analogs. One promising effect of these compounds is their antibacterial and antifungal activity [12,13,14,15,16,17].

The aims of the current study were: (a) to monitor NOS-6 uptake into mouse fibroblasts (BALB/c cells) using liquid chromatography coupled to electrospray ion-trap mass spectrometry (HPLC-ESI/IT-MS), (b) to study the cytotoxicity of NOS-6 using several methods, and (c) to discuss the biological activities of the other cyclopentenediones with structural similarity to NOS-6.

## 2. Results and Discussion

NOS-6 cytotoxicity was tested using mouse fibroblasts. First, we tested the uptake of NOS-6 into cells by HPLC (*t*_R_ 7.1 min) connected on-line with an ESI/IT-MS detector. Identification of NOS-6 in the cell homogenates was based on the following MS^n^ fragmentation pattern, MS (*m/z*) [M+H]^+^ 799.1 → MS^2^ 399.1 → MS^3^ 305.1 → MS^4^ 277.1, which was in good agreement with previously published MS/MS data [10] (Figure 1b). Amounts of 1.5 and 4.5 µg NOS-6 per 1 × 10^5^ cells were found after 4 h incubation where 0.5 and 1 µM NOS-6 were present in the cultivation medium (Figure 1c). On this basis, it was clear that fibroblasts were capable of accumulating NOS-6. For detailed characterization of the uptake, NOS-6 (2.5 µM) was incubated with mouse fibroblasts for 2 h. After termination of the incubation period, 59% of NOS-6 was found in the cytosolic fraction and 41% was bound onto cell membranes (for details see Section 3.4). The mechanism of NOS-6 transport through cell membranes is not known in detail. The transport processes could be facilitated by the spherical shape of NOS-6 (inset in Figure 1c).

**Figure 2:**
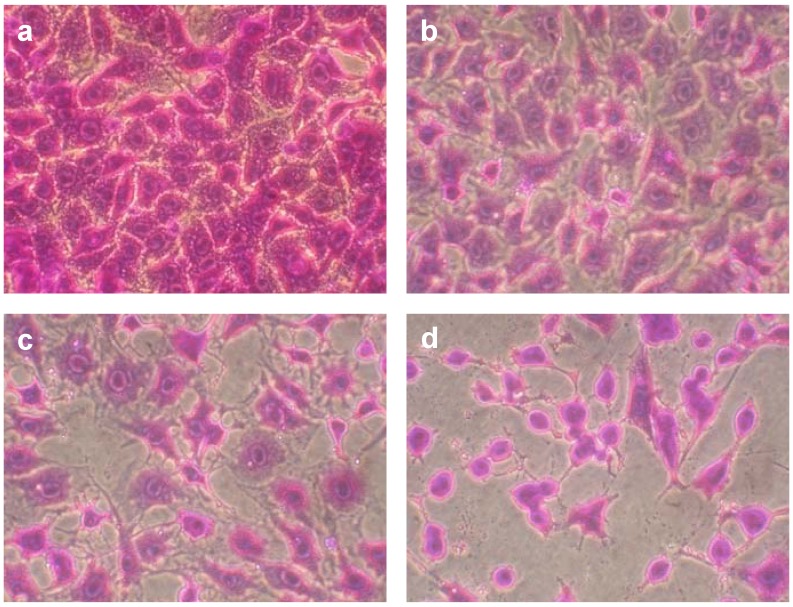
Effect of NOS-6 on mouse fibroblasts (BALB/c cells). Cells were treated with 1, 5 and 10 µM for 24 h (B-D). Control cells (A) were treated with DMSO (0.5%; v/v) for 24 h under the same conditions. Cells were fixed and stained by crystal violet (2% in 20% methanol). Magnification was 400×.

**Figure 3:**
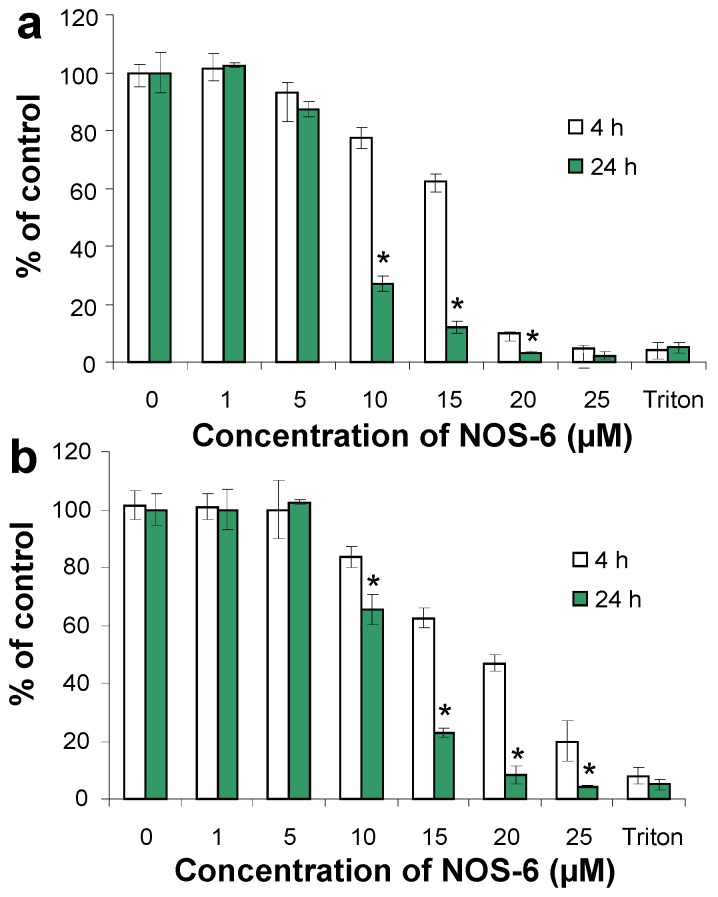
The effect of NOS-6 on the viability of mouse fibroblasts (BALB/c cells). Fibroblasts were treated with NOS-6 (1–25 μM) for 4/24 h; control cells were incubated with DMSO (0.5%; v/v) under the same conditions. Cell damage was evaluated as **(a)** NR retention and **(b)** MTT assay. Data are expressed as percentage of control and plotted as means ± S.D. *) *p* < 0.05 statistically different from 4 h incubation.

**Figure 4:**
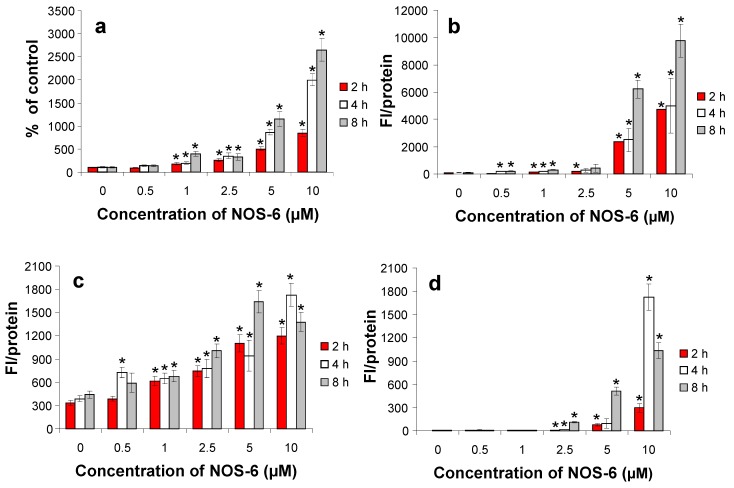
Effects of NOS-6 on **(a)** the cellular membrane integrity measured as LDH in medium and **(b)** caspase 3, **(c)** caspase 8 and **(d)** caspase 9 activities in mouse fibroblasts (BALB/c cells). Fibroblasts were treated with NOS-6 (1–10 μM) for 2/4/8 h; control cells were incubated with DMSO (0.5%; v/v) under the same conditions. Data are presented as means ± S.D. of three independent experiments. FI/protein = Relative Fluorescence Intesity per mg of soluble proteins. (*) *p* < 0.05 statistically different from the untreated cells.

The cytotoxicity examination of NOS-6 was done on mouse fibroblasts over a 4 and/or 24 h cultivation period. The compound was tested in a concentration range from 1 to 25 µM. For concentrations higher than 25 µM, limiting solubility of NOS-6 in cultivation medium was observed. After microscopic observation (Figure 2), biochemical methods based on neutral red retention (NR assay), activity of intracellular dehydrogenases (MTT assay) and level of extracellular lactate dehydrogenase (LDH assay) were used for measuring NOS-6 cytotoxicity (Figure 3 and Figure 4A).

The IC_50_ of NOS-6 after 24 h incubation with fibroblasts was estimated as 8.48 ± 0.16/12.15 ± 1.96 µM (NR/MTT assay). The IC_50_ of NOS-6 was higher than the IC_50_ of doxorubicin – DOX (Table 1). DOX is an anthracycline antibiotic which has been used as a reference agent in the study of the cytotoxic potential of derivatives of cyclopentenedione, coruscanone A [12,14]. 

**Table 1:** Comparison of IC_50_ values for NOS-6 and doxorubicin.

Compound	IC_50_ (µM)	
	NR assay	MTT assay
NOS-6	8.48 ± 0.16	12.15 ± 1.96
Doxorubicin	1.26 ± 0.42	2.04 ± 0.48

This compound isolated from *Piper coruscans* has a structural similarity to one of the cyclopentenedione subunits of NOS-6. The IC_50_ of coruscanone A/vs. DOX after 48 h cultivation was 19.3/13.8 µM (for Vero cells) and 13.4/1.2 µM (for LLC-PK1 cells) [12]. The cyclopentenedione skeleton is also typical for asterredione, a natural compound isolated from *Aspergillus terreus*. The cytotoxicity of asterredione was tested on cancer cell lines, NCI-H460, MCF-7 and SF-268 where the IC_50_ varied between 17 and 25 µM after a 48 h-long experiment [17]. Coruscanones (A/B) and astteredione have already been studied in connection with their antibacterial and antifungal activities. Antibacterial activity was also confirmed for the natural compound G2201-C first isolated from the fermentation broth of *Streptomyces cattleya* [18]. In addition to the naturally occurring cyclopentenediones, the cytotoxicity of synthetic cyclopentenedione derivatives, e.g. the compound TX-1123 (2-hydroxyarylidene-4-cyclopentene-1,3-dione) was studied. IC_50_ of TX-1123 was 57 µM for rat hepatocytes but significantly higher cytotoxicity was found for cancer cell lines. For this reason, TX-1123 is identified as a candidate for potential anticancer therapeutics [19,20].

In the subsequent experiments, we focused on the study of which type of cell death is involved in NOS-6 cytotoxic effects. It was found that NOS-6 can induce both types of cell death, apoptosis and necrosis in a time- and dose-dependent manner. Incubation of cells with higher concentrations of NOS-6 (5 and 10 μM) induced both apoptosis and necrosis (Figure 4). 

Activation of caspases in a dose-dependent manner was observed. The activation of caspase 8 controlled by the death receptor was weaker than caspase 9 controlled by the mitochondrial cascade (Figure 4c, d). Caspase 3 activation results from activation of both cascades controlled by caspase 8 and 9 so that caspase 3 activation is most distinct (Figure 4b). It is possible that pro-apoptotic activity is one of the pathways leading to the cytotoxic effects of NOS-6 in fibroblasts. The apoptosis can be induced using several types of natural compounds under *in vitro* conditions [21]. Caspase 3 was activated after application of cyclopentenediones isolated from *Lindera erythrocarpa*, especially the methyl derivative of lucidone [22].

Inhibition by the cyclopentenediones has been found for a number of enzyme systems. NOS-6 was shown to be an effective inhibitor of acetylcholinesterase and butyrylcholinesterase [9,10,11]. Of structural analogues of NOS-6, TX-1123 is an inhibitor of protein tyrosine kinase [19,20], linderones and lucidones are also capable of inhibiting farnesyl protein transferase [22], and benzylidene cyclopentenediones are inhibitors of the botulinum neurotoxin A’s zinc endopeptidase [23].

## 3. Experimental

### 3.1. Chemicals

Organic solvents for NOS-6 isolation and analysis were obtained from Analytika (Czech Republic), Sigma–Aldrich (St. Louis, MO, USA) and Merck (Darmstadt, Germany). All solutions were prepared using reverse-osmosis deionized water (Ultrapur, Watrex, Prague, Czech Republic). Nitrogen and helium (99.999% for both) were obtained from Linde Gas (Prague, Czech Republic). Dulbecco’s modified Eagle’s medium (DMEM), streptomycin, penicillin, glutamine, fetal calf serum (FCS), newborn calf serum (NCS), trypsin-EDTA (0.25%), MTT (3-(4,5-dimethylthiazol-2-yl)-2,5-diphenyltetrazolium bromide), NADH, doxorubicin hydrochloride and other chemicals were from Sigma–Aldrich, USA. Caspase 3 (Ac-DEVD-AMC/Ac-DEVD-CHO), caspase 8 (Z-IETD-AFC/IETD-CHO) and caspase 9 (Ac-LEHD-AFC/Ac-LEHD-CHO) fluorigenic substrates/inhibitors were obtained from Bachem AG (Switzerland). Protease inhibitor cocktail tablets Complete^TM^ were purchased from Roche Diagnostic (Germany).

### 3.2. Isolation of NOS-6

NOS-6 was isolated from cyanobacterium *Nostoc* sp. strain Lukešová 27/97 obtained from the culture collection of soil algae and cyanobacteria of the Institute of Soil Biology of the Academy of Sciences of the Czech Republic. The cultivation of the cyanobacteria was carried out in Allen & Arnon medium [24]. The isolation of NOS-6 was realized using the methanol/acetone/hexane system according to a previously published protocol [10].

### 3.3. Cell culture

Mouse fibroblasts (BALB/c cells; clone A31) were purchased from the European Collection of Cell Cultures (Salisbury, United Kingdom). Mouse fibroblasts were grown in DMEM supplemented with heat-inactivated fetal calf serum (5%, v/v) and newborn calf serum (5%, v/v), streptomycin (100 U.mL^−1^), penicillin (0.1 mg.mL^−1^) and glutamine (4 mM). Cells were cultured in a humidified atmosphere with 5% (v/v) CO_2_ at 37 °C. For all experiments, BALB/c were seeded in plates at a density of 1 × 10^5^ cells.cm^-2^ and grown to near confluence for 1 day.

### 3.4. Identification of NOS-6 and its uptake into mouse fibroblasts

HPLC with quadrupole ion-trap (IT) MS detector LCQ Fleet (Thermo Scientific, Waltham, MA, USA) operating in a positive ESI mode was used for NOS-6 analysis in mouse fibroblasts. Following the treatment period, the fibroblasts (1 × 10^7^ cells per sample) were scraped off the cultivation dish and separated by centrifugation (20×*g*, 10 min, 4 °C, Eppendorf, Hamburg, Germany). The medium was removed and stored for HPLC-ESI/IT-MS analysis. The cells were washed three times by PBS and centrifuged (20×*g*, 10 min, 4 °C). The cells resuspended in PBS (250 µL) were destroyed by the freezing/thawing cycle (–80 °C, three times) and were sonicated using homogenizer UP 200S (cycle 0.5, amplitude 50, 30-times) from Hielscher Ultrasonics GmbH (Teltow, Germany). The cell lysate was centrifuged (14 000×*g*, 10 min, 4 °C) and the supernatant (cytosolic fraction) was used for HPLC-ESI/IT-MS analysis of NOS-6 uptake into cells. The sediment was washed three times by PBS, resuspended in lysis solution (0.02% Triton X-100; 0.1% sodium dodecyl sulfate in PBS) and sonicated (cycle 0.5, amplitude 50, 30-times). After 10 min incubation (4 °C) the suspension was centrifuged (14 000×*g*, 10 min, 4 °C) and the supernatant was used for HPLC-ESI/IT-MS analysis of NOS-6 bound to the membranes. Finally, all samples were mixed with methanol/4% aqueous acetic acid (70/30; %, v/v) in a ratio of 1:1 (v/v), centrifuged (14 000×*g*, 10 min, 4 °C) and supernatants (10 µL) were analyzed by HPLC-ESI/IT-MS.

The HPLC chromatographic system (Shimadzu, Kyoto, Japan) equipped with SCL-10Avp controller, a vacuum degasser, a binary pump (LC-10ADvp), an autoinjector (SIL-10ADvp), a column oven (CTO-10ACvp) and a UV-detector (SPD10Avp) was used. The system was coupled on-line to an ESI/IT MS detector. The chromatographic column used was Eclipse XDB-CN from Agilent (USA), 150 mm × 2.1 mm, 5 µm. The injection volume was 10 µl and the mobile phase consisted of methanol (solvent B)/2% acetic acid in 10% methanol aqueous solution, linear gradient elution: 0–8 min (40–80% B), 8–8.1 min (80–40% B), and 8.1–10 min (40% B). The mobile phase flow rate was 0.4 mL.min^−1^, and the temperature of the column oven was set at 30 °C.

The ESI-MS parameters were as follows: spray voltage (4.75 kV), capillary temperature (300 °C) and capillary voltage 20 V. Nitrogen was used as sheath, auxiliary, and sweep gas, and helium was used as the collision gas. The sheath, auxiliary, and sweep gas flow rates were 50, 5, and 1 (as arbitrary units), respectively. Full-scan mass spectra were acquired in the range 50–1,000 *m/z*. The abundance of MS^2^ fragment of NOS-6 was monitored for analysis in real samples.

### 3.5. Cytotoxicity of NOS-6

Stock solutions of NOS-6 (0.1–5.0 mM) were dissolved in DMSO. To determine test compound cytotoxicity, cells were treated with NOS-6 (0.5–25 µM) in serum-free medium for 2, 4, 8 and 24 hours. Control cells were treated only in serum-free medium with an aliquot of DMSO instead of test compound. The final percentage representation of DMSO in the medium was 0.5%. Cells were treated with serum free medium containing 0.5% of Triton X-100 to evaluate maximal toxicity. After incubation, the following parameters of cell damage were assessed for evaluation of test compound cytotoxicity: cell viability (NR assay), activity of intracellular dehydrogenases (MTT assay), level of extracellular lactate dehydrogenase (LDH assay) [25] and caspase 3, 8 and 9 [26]. DOX toxicity (IC_50_, μM) was determined according to the same protocols as NOS-6. The stock solutions of DOX were prepared in sterile deionized water.

### 3.6. Statistical analysis

Three/four separate experiments were performed in three replicates for each sample. Data were expressed as means ± S.D. Statistical comparison between untreated cells and cells treated with test compound was performed using Student’s t-tests.

## 4. Conclusions

This study describes the cytotoxicity and pro-apoptotic activity of NOS-6 on mouse fibroblasts BALB/c. NOS-6 is a naturally occurring polyphenol derived from bis(cyclopentenedione) and its cytotoxicity was evaluated using several cell viability assays. The IC_50_ of NOS-6 after 24 h incubation was found to be 8.48 ± 0.16/12.15 ± 1.96 µM (NR/MTT assay). The cytotoxicity of NOS-6 was lower than that of DOX. At lower doses, the main mechanism of NOS-6 cytotoxicity is probably linked to induction of apoptosis and this was confirmed by measurement of activities of selected caspases in mouse fibroblasts. The higher doses of NOS-6 induced necrosis. This pilot study provides a solid base for further study of the cytotoxicity and biological activity of the cyclopentenediones and their hydroxyphenyl derivatives (e.g. [27,28]).
